# From Suppurative Inflammation to Fibrotic Remodeling in Cutaneous *Nocardiopsis* Infection

**DOI:** 10.4269/ajtmh.26-0141

**Published:** 2026-08-25

**Authors:** Naixuan Lin, Keke Huang, Degang Yang

**Affiliations:** Department of Infectious Dermatosis, Center of Infectious Skin Diseases, Shanghai Skin Disease Hospital, Tongji University School of Medicine, Shanghai, China

## Abstract

In the present case report, a cutaneous infection caused by the rare actinomycete *Nocardiopsis* sp. TSRI0078 is described. A 77-year-old woman with a medical history of hypertension and diabetes presented with ulcerated purplish-red nodules on the lower extremities that had persisted for 3 months. A histopathological examination revealed infectious granulomas predominantly composed of neutrophils. After empirical antibiotic therapy proved ineffective, the pathogen was confirmed to be *Nocardiopsis* sp. TSRI0078 via polymerase chain reaction testing, gene sequencing, and microbial culture. Subsequent treatment with oral trimethoprim–sulfamethoxazole led to gradual regression of the lesions and scar formation. With only 11 human infection cases reported globally, a clear understanding of the clinical manifestations of *Nocardiopsis* infections is lacking. This pathogen should be considered in the differential diagnosis of chronic, unexplained cutaneous lesions, particularly in patients with underlying diseases. A combination of molecular and conventional microbiological examinations facilitates early diagnosis, and trimethoprim–sulfamethoxazole may represent an effective therapeutic option.

## INTRODUCTION

*Nocardiopsis* is phylogenetically related to *Nocardia* and was first described and classified by Meyer in 1976. Both genera belong to the order *Actinomycetales* and constitute a distinct and coherent lineage within the bacterial taxonomic system.^1^ Although most *Nocardiopsis* species are considered nonpathogenic, a few have been recognized as causative agents of human infection. Here, we report a case of cutaneous *Nocardiopsis* sp. TSRI0078 infection, prepared in accordance with the CARE guidelines (Supplemental Table 1). Only 11 cases of *Nocardiopsis* infection have been documented worldwide so far, primarily affecting the skin or subcutaneous tissues, respiratory tract, nasal cavity, bloodstream, and ocular structures, with the majority occurring in immunocompromised individuals or those with underlying medical conditions (Supplemental Table 2).^2^ The pathogenic potential of *Nocardiopsis* is associated with its capacity for invasive growth, immune evasion, toxin production, and biofilm formation.^3^

*Nocardia* and *Actinomycetes* are common etiological agents of mycetoma that may exhibit distinct histopathological patterns: *Actinomycete*-related mycetoma typically presents as a chronic granulomatous infection with prominent fibrosis, whereas *Nocardia*-associated mycetoma is characterized by a purulent inflammatory response.^4^^,^^5^ A case of cutaneous multiple nodules caused by *Nocardiopsis* sp. TSRI0078 infection is reported in the present study, with a discussion on its clinical features, diagnostic approaches, and therapeutic management to inform clinical practice.

## CASE PRESENTATION

The patient was a 77-year-old retired woman who lived with her husband in a standard residential apartment in an urban area of Shanghai. Her sanitation and hygiene practices were adequate, and she had no history of agricultural work, soil exposure, or animal contact. The patient presented with a “skin rash on both lower limbs accompanied by pain for more than three months.” Three months before presentation, after the patient experienced trauma to the right lower limb, cutaneous nodules developed and progressively increased in size and number, eventually involving both lower limbs, with associated ulceration and pain. She had a medical history of hypertension and diabetes mellitus for ∼17 years, which were managed with long-term oral medications, although without regular monitoring of disease control. A physical examination revealed scattered, asymmetrically distributed purplish-red nodules on both lower limbs, approximately the size of broad beans, some exhibiting surface ulceration; superficial lymphadenopathy was not detected (Figure 1A).

**Figure 1.**
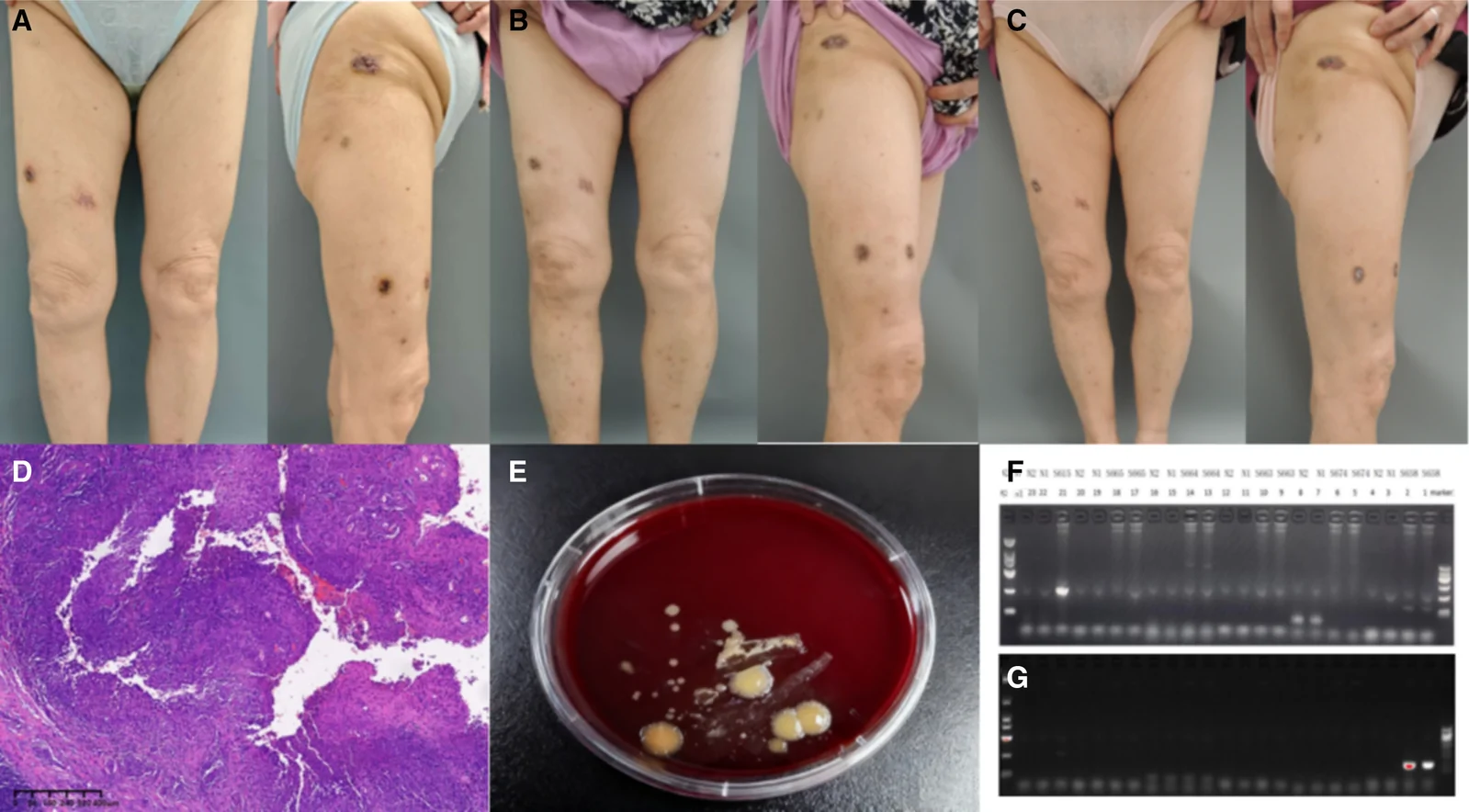
(**A**) Initial presentation revealing scattered purplish-red nodules on both lower limbs, some ulcerated with sinus tracts. (**B**) After 4 weeks of trimethoprim–sulfamethoxazole treatment, nodules contracted and healed. (**C**) After 4 months of treatment, lesions exhibited fibrotic changes with scar formation. (**D**) Histopathology revealing an infectious granuloma with neutrophil infiltration (hematoxylin and eosin stain, 400×). (**E**) Pale yellow colonies grown on Middlebrook 7H10/7H11 agar at 28°C for 2 weeks. (**F**) Polymerase chain reaction amplification results from skin lesion secretions. (**G**) Polymerase chain reaction amplification results from cultured colonies.

The patient first presented to the study hospital on February 24, 2025, with initial differential diagnoses including vasculitis, vascular tumor, or disseminated infection (the diagnostic flowchart for this case is summarized in Supplemental Figure 1). Laboratory evaluations including complete blood count, C-reactive protein levels, liver and renal function tests (Supplemental Table 3), T-cell spot of tuberculosis, fungal culture, and nucleic acid testing for nontuberculous mycobacteria revealed no significant abnormalities. Between March and May 2025, three biopsies were performed, all of which revealed infectious granulomas predominantly infiltrated by neutrophils (Figure 1D). Periodic acid–Schiff staining, acid-fast staining, Giemsa staining, and Gram staining results were all negative (Supplemental Figure 2). Immunohistochemical staining results for CD34, CD31, and E26 transformation-specific (ETS)–related gene were also negative.

After the first biopsy, empirical anti-infective therapy with moxifloxacin in combination with clarithromycin was initiated. After the second biopsy, the original treatment regimen was maintained. During the third biopsy, metagenomic next-generation sequencing (mNGS) was performed on tissue from a clean skin lesion, which suggested *Pseudomonas putida*. However, the lesions continued to progress despite treatment. On May 21, 2025, the laboratory conducted conventional polymerase chain reaction (PCR) amplification and sequencing of secretions from the skin lesion, which indicated *Nocardiopsis* infection (Figure 1F). Concurrently, aerobic culture of the secretions at 28°C for 2 weeks yielded pale yellow, wrinkled colonies (Figure 1E). Subsequent PCR amplification and BLAST analysis confirmed the isolate as *Nocardiopsis* sp. TSRI0078 (Figure 1G). On the basis of the Sanford Guide to Antimicrobial Therapy and Infectious Diseases Society of America-related guidelines, the treatment was changed to oral trimethoprim–sulfamethoxazole (480 mg, twice daily) starting on June 11, 2025.^6^^,^^7^ Follow-up revealed gradual regression of the nodules and formation of hypopigmented scars, indicating definite therapeutic efficacy (Figure 1B and C).^6^^,^^7^

## DISCUSSION

The histopathological spectrum of mycetoma varies according to the causative pathogen. Mycetoma typically presents pathologically as granulomatous inflammation, with common etiological agents including *Nocardia*, *Nocardiopsis*, *Actinomycetes*, and fungi.^8^ Early-stage infectious granulomas are predominantly driven by neutrophils, intermediate stages are characterized by macrophage dominance, and late stages involve chronic remodeling (Supplemental Figure 3).^9^^,^^10^ The prominence of neutrophil-mediated suppurative lesions versus fibrosis-mediated scarring differs across specific diseases.

The dynamic evolution of a single lesion in this patient provides direct observational evidence for understanding the natural progression of granulomatous inflammation caused by *Nocardiopsis* infection. The lesion on the proximal right lower limb exhibited distinct histopathological stages: initially presenting with ulceration and purulent exudate, histology revealed abundant neutrophil infiltration; after treatment, the nodules became nonulcerated with features of granulation tissue; subsequently, the nodules regressed, leaving fibrotic scars, corresponding histologically to fibroproliferation and tissue remodeling. This continuous progression supports the characterization of Nocardiopsis-associated cutaneous infection as suppurative inflammation coexisting with granulomatous reaction.^11^ Although mNGS of the lesion tissue suggested *Pseudomonas putida*, this finding was inconsistent with the clinical course and likely represents colonization or contamination because *Pseudomonas putida* is frequently encountered as a contaminant or colonizer in clinical samples and typically causes acute cellulitis, a presentation distinct from the chronic granulomatous lesions observed in the patient.^12^^,^^13^ The lesion’s evolutionary pattern aligns with the dynamic development of granulomas, clearly illustrating the local immune response to *Nocardiopsis*—from acute neutrophil-mediated suppuration, transitioning to chronic granuloma formation, and ultimately progressing to fibrotic remodeling (Supplemental Figure 4).

Notably, long-standing diabetes mellitus in this patient served as a critical predisposing factor for the development of this opportunistic cutaneous infection. Hyperglycemia promotes cutaneous infections in diabetes through two synergistic mechanisms. First, the glucose-rich microenvironment in the skin provides an abundant carbon source that fuels rapid pathogen proliferation. Under antibiotic pressure, this metabolic advantage also favors the emergence of drug-resistant mutants, thereby exacerbating the infection.^14^ Second, hyperglycemia induces glucose transporter 2 (GLUT2)-dependent transcriptional reprogramming in epithelial cells, which disrupts tight junctions and adherens junctions. This disruption increases the permeability of the skin barrier and facilitates the translocation of microorganisms and their metabolites. In diabetes, these barrier defects are often accompanied by impaired phagocytic function, creating an ideal niche for opportunistic bacteria that rely on compromised tissue barriers and weakened immune clearance.^15^ Consequently, Nocardiopsis species, typically low-virulence environmental actinomycetes, can invade, colonize, and persist, which explains the heightened susceptibility of diabetic patients to such infections.

## CONCLUSION

*Nocardiopsis* is a rare opportunistic pathogen capable of causing chronic suppurative granulomatous infections of the skin and subcutaneous tissues.^16^^,^^17^ Its pathological features lie between those of *Actinomycetes* and *Nocardia*, and in mycetoma, it may elicit mixed inflammatory reactions, occasionally accompanied by granule formation. Clinically, in cases of chronic skin lesions of unknown etiology, particularly among patients with underlying conditions or compromised immune function, *Nocardiopsis* infection should be considered in the differential diagnosis. Early diagnosis hinges on recognition of characteristic clinical manifestations, integrated with histopathological and microbiological examinations, and is supported by molecular biological techniques,^18^^,^^19^ thereby facilitating targeted anti-infective therapy and improving patient outcomes.

## Supplemental Materials

10.4269/ajtmh.26-0141 Supplemental Materials
