# Supplementary material for: From Suppurative Inflammation to Fibrotic Remodeling in Cutaneous Nocardiopsis Infection
Source: Am J Trop Med Hyg. 2026 Aug 25;115(4):721–3. doi: 10.4269/ajtmh.26-0141 (PMC13527196; doi:10.4269/ajtmh.26-0141)
Supplement: Supplementary file 1 [file tpmd260141.SD1.pdf]

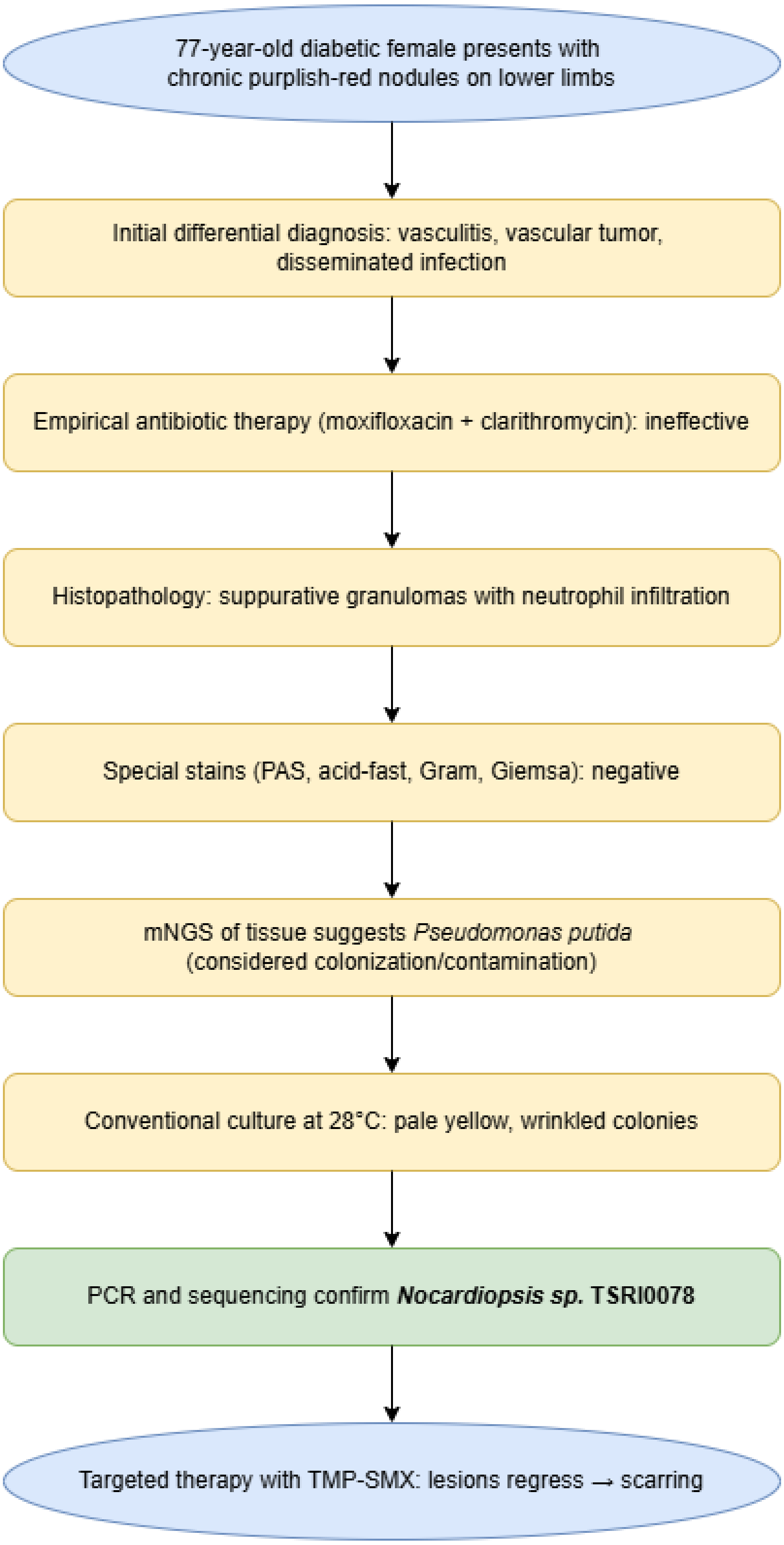


**Figure S1.** Flowchart showing the diagnostic process from initial presentation to confirmed Nocardiopsis infection, including clinical findings, histopathology, culture, molecular identification, and successful TMP-SMX treatment.

**Figure S2. Special stains of the skin lesion.**

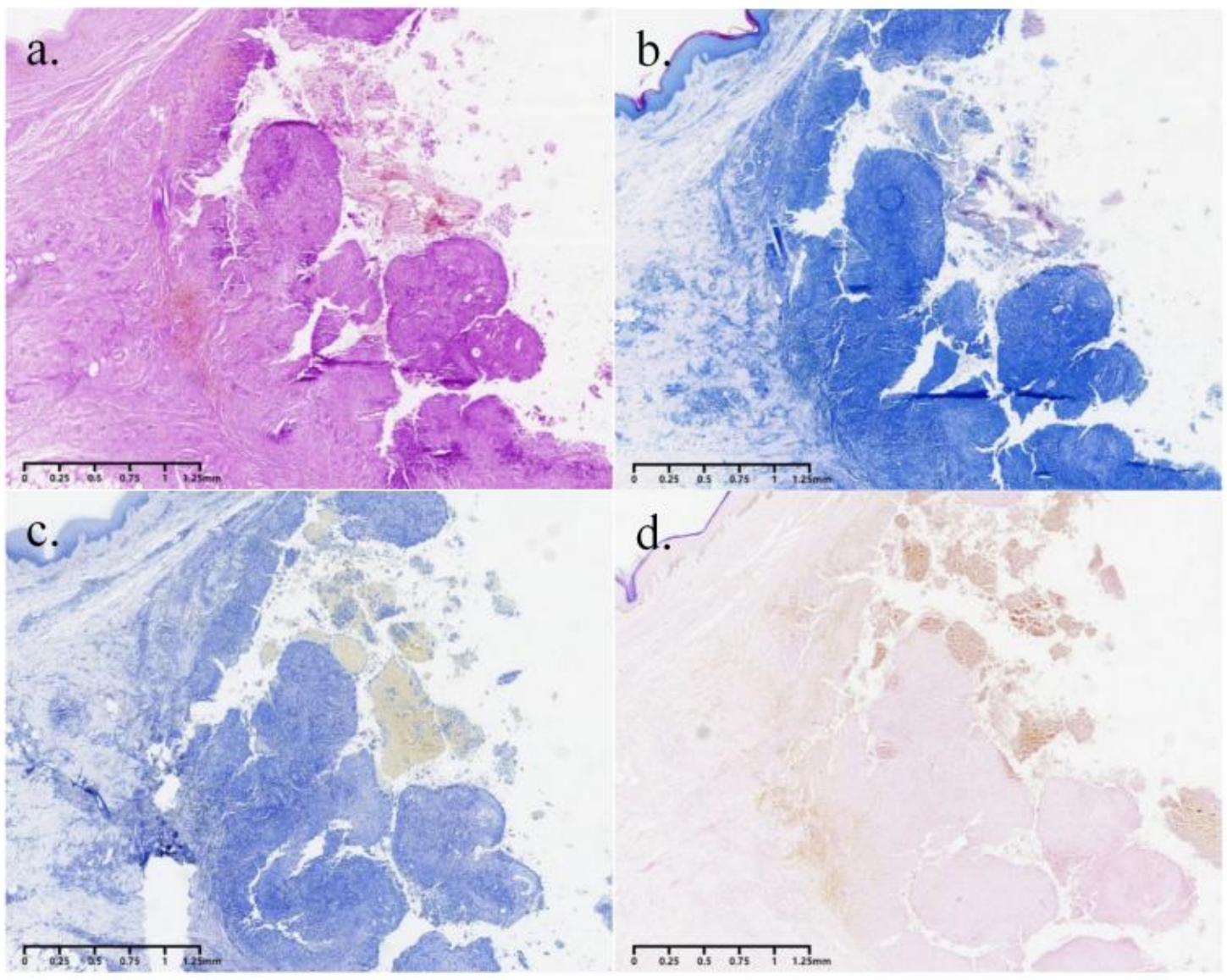


**Figure S2.** Special stains of the skin lesion. (**A**) Periodic acid–Schiff (PAS) stain, negative. (**B**) Kinyoun's acid-fast stain, negative. (**C**) Giemsa stain, negative. (**D**) Gram stain, negative.

**Figure S3. Stages of granuloma formation in cutaneous infection.**

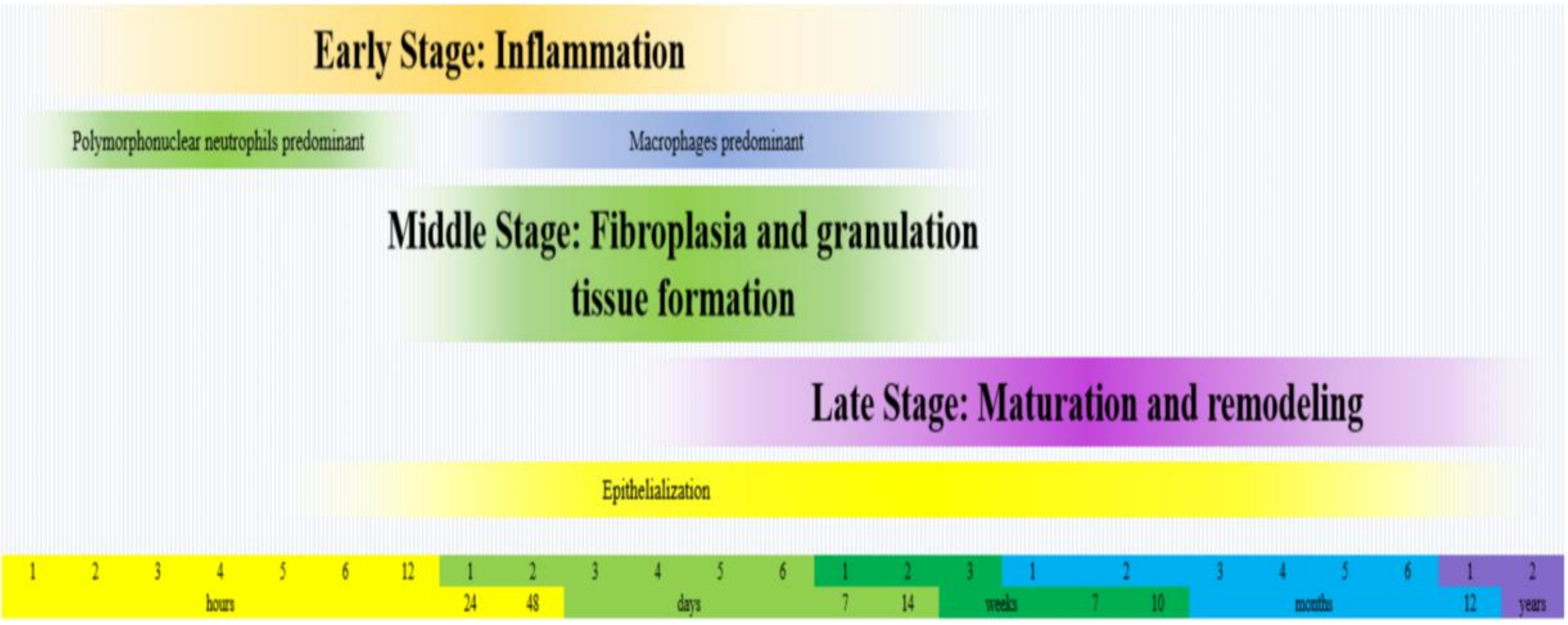


**Figure S3.** Schematic diagram illustrating the three stages of granuloma formation in cutaneous infection: early stage with neutrophil predominance, intermediate stage with macrophage predominance, and late stage with chronic remodeling and fibrosis.

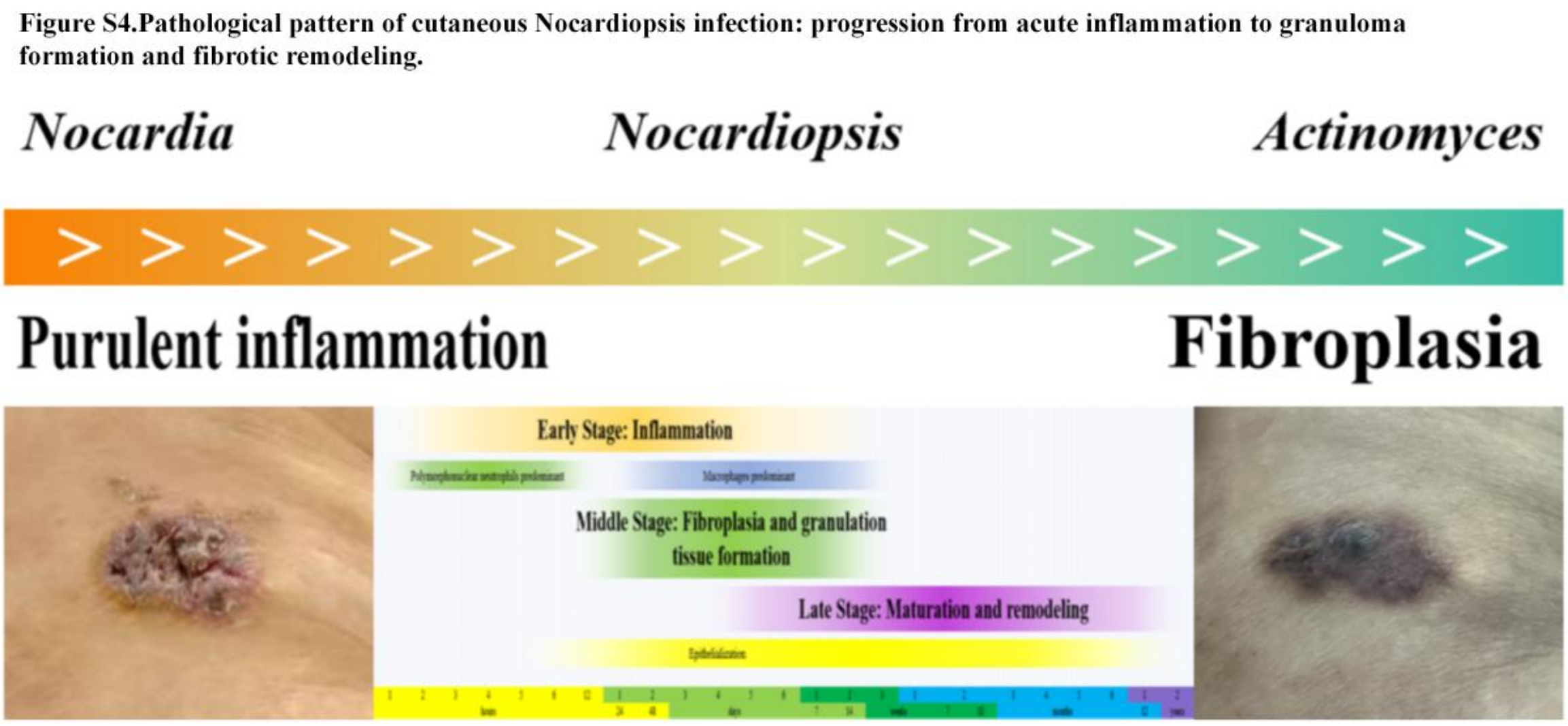


**Figure S4.** Pathological pattern of cutaneous Nocardiopsis infection: progression from acute inflammation to granuloma formation and fibrotic remodeling.

CARE
case report guidelines

# CARE Checklist of information to include when writing a case report

(cc) BY-NC-ND

| Topic | Item | Checklist item description | Reported on Line |
|---|---|---|---|
| Title | 1 | The diagnosis or intervention of primary focus followed by the words "case report" | 1 |
| Key Words | 2 | 2 to 5 key words that identify diagnoses or interventions in this case report, including "case report" | 25 |
| Abstract (no references) | 3a | Introduction: What is unique about this case and what does it add to the scientific literature? | 14-18 |
| | 3b | Main symptoms and/or important clinical findings | 19-23 |
| | 3c | The main diagnoses, therapeutic interventions, and outcomes | 19-23 |
| | 3d | Conclusion—What is the main "take-away" lesson(s) from this case? | 24-26 |
| Introduction | 4 | One or two paragraphs summarizing why this case is unique (**may include references**) | 179-188 |
| Patient Information | 5a | De-identified patient specific information. | 40 |
| | 5b | Primary concerns and symptoms of the patient. | 40 |
| | 5c | Medical, family, and psycho-social history including relevant genetic information | 40 |
| | 5d | Relevant past interventions with outcomes | 46-50 |
| Clinical Findings | 6 | Describe significant physical examination (PE) and important clinical findings. | 40-48 |
| Timeline | 7 | Historical and current information from this episode of care organized as a timeline | 40-50 |
| Diagnostic Assessment | 8a | Diagnostic testing (such as PE, laboratory testing, imaging, surveys). | 42-48 |
| | 8b | Diagnostic challenges (such as access to testing, financial, or cultural) | 46-48 |
| | 8c | Diagnosis (including other diagnoses considered) | 42-48 |
| | 8d | Prognosis (such as staging in oncology) where applicable | 50 |
| Therapeutic Intervention | 9a | Types of therapeutic intervention (such as pharmacologic, surgical, preventive, self-care) | 46-50 |
| | 9b | Administration of therapeutic intervention (such as dosage, strength, duration) | 50 |
| | 9c | Changes in therapeutic intervention (with rationale) | 46-50 |
| Follow-up and Outcomes | 10a | Clinician and patient-assessed outcomes (if available) | 50 |
| | 10b | Important follow-up diagnostic and other test results | 46-50 |
| | 10c | Intervention adherence and tolerability (How was this assessed?) | 179-188 |
| | 10d | Adverse and unanticipated events | 179-188 |
| Discussion | 11a | A scientific discussion of the strengths AND limitations associated with this case report | 90-108 |
| | 11b | Discussion of the relevant medical literature **with references**. | 90-109 |
| | 11c | The scientific rationale for any conclusions (including assessment of possible causes) | 90-108 |
| | 11d | The primary "take-away" lessons of this case report (without references) in a one paragraph conclusion | 90-108 |
| Patient Perspective | 12 | The patient should share their perspective in one to two paragraphs on the treatment(s) they received | 179-188 |
| Informed Consent | 13 | Did the patient give informed consent? Please provide if requested | Yes ☑ No ☐ |

**Table S2. Demographic, clinical and therapeutic characteristics of all documented human *Nocardiopsis* infection cases**

| Case No. | Year | Region | Gender | Age (years) | Underlying disease / Immune status | Infection site | Pathogen | Identification method | Treatment regimen | Outcome | Reference |
|---|---|---|---|---|---|---|---|---|---|---|---|
| 1 | 1985 | North America | Male | 39 | Local trauma history | Cutaneous | *N. dassonvillei* | Morphological and biochemical tests | Trimethoprim-sulfamethoxazole | Improved | [1] |
| 2 | 1998 | Europe | Male | 46 | Chronic pneumonia | Pulmonary | *N. dassonvillei* | Chemotaxonomic tests + DNA-DNA hybridization | Amikacin | Improved | [2] |
| 3 | 1999 | Europe | Male | 60 | Arterial hypertension | Bloodstream | *N. dassonvillei* | 16S rRNA gene sequencing | Piperacillin + ciprofloxacin | Cured | [3] |
| 4 | 2005 | Asia | Male | 3 | No chronic disease | Bloodstream | *N. dassonvillei* | Morphological and biochemical tests | Trimethoprim + gentamicin | Improved | [4] |
| 5 | 2011 | Europe | Male | 57 | Long-term low-dose prednisone; extrinsic allergic alveolitis | Cutaneous | *N. dassonvillei* | 16S rRNA gene sequencing | Trimethoprim-sulfamethoxazole | Cured | [5] |
| 6 | 2012 | Asia | Male | 55 | Diabetes | Nasal vestibule | *N. dassonvillei* | 16S rRNA gene sequencing | Trimethoprim-sulfamethoxazole + clarithromycin + levofloxacin | Cured | [6] |
| 7 | 2020 | Europe | Male | 85 | COPD; long-term inhaled corticosteroid | Pulmonary | *N. dassonvillei* | 16S rDNA gene sequencing | Cefotaxime + amikacin + metronidazole | Died | [7] |
| 8 | 2022 | Asia | Female | 53 | No chronic disease | Cutaneous | *Nocardiopsis spp.* | 16S rRNA gene sequencing | Trimethoprim-sulfamethoxazole + levofloxacin | Cured | [8] |
| 9 | 2022 | Asia | Male | 69 | Cardiac valve disease | Disseminated | *Nocardiopsis spp.* | Metagenomic sequencing | Ceftriaxone + linezolid | Cured | [9] |
| 10 | 2023 | Asia | Female | 35 | No chronic disease | Cutaneous | *N. dassonvillei* | DNA molecular detection | Thread removal + local 5- FU + triamcinolone acetonide | Cured | [10] |
| 11 | 2024 | Asia | Male | 65 | Pulmonary adenocarcinoma; chronic bronchitis with emphysema | Pulmonary | *N. dassonvillei* | mNGS | Trimethoprim-sulfamethoxazole + moxifloxacin | Improved | [11] |

COPD, chronic obstructive pulmonary disease; mNGS, metagenomic next-generation sequencing.

**References for Supplementary Materials**

[1] Sindhuphak W, Macdonald E, Head E. Actinomycetoma caused by Nocardiopsis dassonvillei. Arch Dermatol. 1985;121(10):1332-1334. PMID: 4037831

[2] Mordarska H, Zakrzewska-Czerwińska J, Paściak M, et al. Rare, suppurative pulmonary infection caused by Nocardiopsis dassonvillei. FEMS Immunol Med Microbiol. 1998;21(1):47-55. doi:10.1111/j.1574-695X.1998.tb01148.x. PMID: 9657320

[3] Beau F, Bollet C, Coton T, et al. Molecular identification of a Nocardiopsis dassonvillei blood isolate. J Clin Microbiol. 1999;37(10):3366-3368. doi:10.1128/JCM.37.10.3366-3368.1999. PMID: 10488208

[4] Lejbkowicz F, Kudinsky R, Samet L, Belavsky L, Barzilai M, Predescu S. Identification of Nocardiopsis dassonvillei in a blood sample from a child. Am J Infect Dis. 2005;1:1-4.

[5] González-López MA, González-Vela MC, Salas-Venero CA, Conde R, Val-Bernal JF. Cutaneous infection caused by Nocardiopsis dassonvillei presenting with sporotrichoid spread. J Am Acad Dermatol. 2011;65(3):e90-e91. doi:10.1016/j.jaad.2011.05.006

[6] Shivaprakash MR, Sumangala B, Prasanna H, et al. Nasal vestibulitis due to Nocardiopsis dassonvillei in a diabetic patient. J Med Microbiol. 2012;61(Pt 8):1168-1173. doi:10.1099/jmm.0.038240-0

[7] Scussel R, Lotte R, Gillon J, Chassang M, Boudoumi D, Ruimy R. Fatal pulmonary infection related to Nocardiopsis dassonvillei in a patient with chronic obstructive pulmonary disease. New Microbes New Infect. 2020;35:100654. Published 2020 Feb 11. doi:10.1016/j.nmni.2020.100654

[8] Lin ZQ, Liu JF, Zhu Y, Chen TT. Nocardiopsis: a case report and literature review [published in Chinese]. Zhonghua Gan Ran He Hua Liao Za Zhi. 2022;22(1):46-50. doi:10.16718/j.1009-7708.2022.01.009.

[9] Zhang XJ, Pan YT, Fan L, Zhang W. A case of disseminated nocardiopsis [Article in Chinese]. Chin J Infect Chemother. 2022;22(5):607-608.

[10] Jia X, Huang L, Hu J. A Rare Symptom of Secondary Infection After Facial Thread Lift and Its Treatment. J Craniofac Surg. 2023;34(2):e204-e205. doi:10.1097/SCS.0000000000009175

[11] He Q, Guan QC, Zhu GF, et al. Lung cancer complicated with Nocardiopsis dassonvillei pulmonary infection: a case report [Article in Chinese]. J Mod Med Health. 2024;40(17):2942-2944, 2947.

**Table S3. Clinical and laboratory parameters of the patient at initial presentation**

| Parameter | Result | Reference range |
|---|---|---|
| Inflammatory marker | | |
| C-reactive protein (CRP) (mg/L) | 8.2 | < 10 |
| Complete blood count | | |
| White blood cell count ($\times 10^9$/L) | 6.5 | 3.5–9.5 |
| Hemoglobin (g/L) | 122 | 115–150 |
| Platelet count ($\times 10^9$/L) | 238 | 125–350 |
| Glucose metabolism | | |
| Fasting glucose (mmol/L) | 8.6 | 3.9–6.1 |
| Postprandial glucose (mmol/L) | Not done | < 7.8 |
| HbA1c (%) | 8.9 | 4.0–6.0 |
| Lipid profile | | |
| Total cholesterol (mmol/L) | 5.8 | < 5.2 |
| Triglycerides (mmol/L) | 2.1 | < 1.7 |
| HDL cholesterol (mmol/L) | 0.9 | > 1.0 |
| LDL cholesterol (mmol/L) | 3.6 | < 3.4 |
| Liver function tests | | |
| Alanine aminotransferase (ALT) (U/L) | 24 | 7–40 |
| Aspartate aminotransferase (AST) (U/L) | 26 | 13–35 |
| Total bilirubin (μmol/L) | 11.5 | 3.4–20.5 |
| Kidney function tests | | |
| Serum creatinine (μmol/L) | 70 | 41–81 |
| Blood urea nitrogen (BUN) (mmol/L) | 5.6 | 2.6–7.5 |
| Estimated GFR (mL/min/1.73m$^2$) | 76 | ≥ 90 |
